# COVID-19 BBIBP-CorV vaccine and transient heart block – A phenomenon by chance or a possible correlation – A case report

**DOI:** 10.1016/j.amsu.2021.102956

**Published:** 2021-10-15

**Authors:** Pirbhat Shams, Jamshed Ali, Sheema Saadia, Aamir Hameed Khan, Fateh Ali Tipoo Sultan, Javed Tai

**Affiliations:** Section of Cardiology, Department of Medicine, The Aga Khan University, Karachi, Pakistan

**Keywords:** Case report, Arrythmia, COVID19, Humoral, Heart block, Atrioventricular block, CIED

## Abstract

**Importance:**

Bradyarrhythmia during COVID19 illness carries prognostic significance. Electrophysiological side effects of COVID19 vaccine remain largely unknown. It is imperative to report nature of cardiovascular side effects of the vaccine.

**Case presentation:**

An 80 years-old-man presented with complains of dizziness, trepidation and shortness of breath following his first shot of COVID-19 BBIBP-CorV (Sino-pharm). ECG on arrival showed 2:1 atrioventricular block with an underlying old left bundle branch block. The AV block changed into Mobitz type-I over the course of next 2 days and into a sinus 1:1 conduction on fourth day of presentation. However, our patient underwent permanent pacemaker implantation due to the underlying conduction tissue disease and intermittent 2:1 AV block during the hospital stay.

**Clinical discussion:**

It is likely that patients with an already diseased conduction system are at an increased risk of worsening of AV block following inoculation of the vaccine. Vaccine associated AV blocks are likely to be reversible. Presence of prior coronary artery disease and electrical abnormalities are important considerations.

**Conclusion:**

COVID-19 vaccine may have added side effects in subjects with known heart disease. Humoral response towards the vaccine might interfere with the conduction system of the heart and more so in patients with diseased and scarred myocardium.

## Highlights

• Patients with underlying existing conduction tissue disease might be at an increased risk of worsening of AV block following inoculation of BBIBP-CorV vaccine.
• Decision to implant permanent pacemaker should be indivisualised based on presence of prior coronary artery disease, conduction tissue abnormality, overall life expectancy and symptom correlation.

## 1 Introduction

The association of COVID-19 illness and bradyarrhythmia has been well reported during the pandemic [[1], [2], [3], [4]]. Bradyarrhythmia during COVID-19 illness carries prognostic significance [5]. Cytokine/inflammatory storm, hypoxia, electrolyte imbalances, and myocardial injury is thought to contribute to the arrhythmogenesis during the illness [6].

The AstraZeneca vaccine analysis report published on April 01, 2021, has reported few heart blocks, the details of which are not known [7]. The literature is silent over any possibility of AV block following COVID-19 BBIBP-CorV. In this report, we present a case of a transient worsening of conduction block following COVID-19 vaccination (BBIBP-CorV) in an 80-year-old-man who had prior coronary artery disease.

The case report has been reported in line with the SCARE 2020 criteria [8].

## 2 Case presentation

An 80-year-old-man presented with complains of multiple episodes of dizziness and trepidation for 3 days and shortness of breath for one day. On arrival, patient had blood pressure of 160/100 mmHg, heart rate of 46 beats per minute (BPM), respiratory rate of 19 breaths per minute and oxygen saturation of 94% on room air. Physical examination was suggestive of a regular pulse and bi-basal chest crackles. There was no postural drop or neurological deficit. There were no signs of peripheral hypoperfusion such as cold peripheries, reduced urine output, or altered mentation. Precordial examination revealed no added sound or murmur. All peripheral pulses were palpable and equal. The patient was known hypertensive and had history of coronary artery bypass grafting (CABG) 18-years ago. Drug history included: Aspirin, Ivabradine and amlodipine (unchanged for last many years; no regular follow-up with cardiologist). Patient had no known addictions and psychosocial history was insignificant. Patient has had no previous similar episodes. Five days before the presentation, patient had received his first shot of BBIBP-CorV (Sino-pharm).

**Fig. 1:**
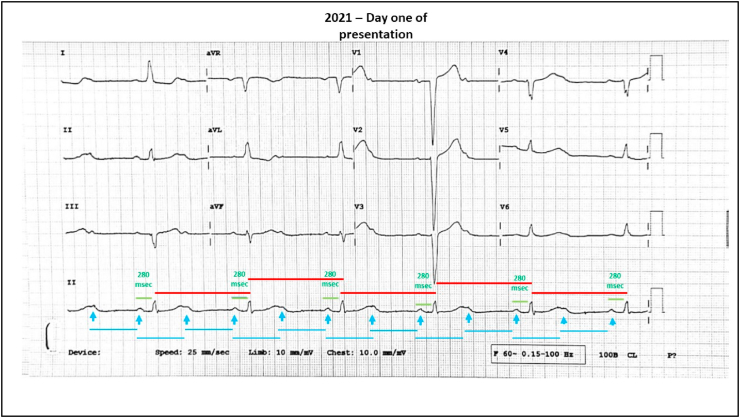
Electrocardiogram on arrival showing 2:1 atrioventricular (AV) block with an atrial rate of approximately 75 BPM and a ventricular rate of approximately 38 BPM. The PR interval was 280 msec. The ECG showed old LBBB.

Electrocardiogram (ECG) on arrival showed 2:1 atrioventricular (AV) block with an atrial rate of approximately 75 BPM and a ventricular rate of approximately 38 BPM. The PR interval of conducted P wave was 280 msec and there was an underlying old left bundle branch block (LBBB) (Fig. 1). Laboratory investigation revealed a hemoglobin of 12 g/dl, white cell count of 8.5 *10^9, Troponin-I of 0.03 ng/ml (cut off 0.04), creatinine of 0.9 mg/dl (cutoff 1.3), Sodium of 121 mmol/L (cutoff 136), Potassium of 4.1 mmol/L, bicarbonate of 21.4 mmol/L (cutoff 21), C-reactive protein of 20 mg/L, magnesium of 1.7 mg/dL (cut off 1.6), and calcium of 8.5 mg/dL (cutoff 8.6). Nasopharyngeal COVID-19 PCR was negative. Patient had a thyroid stimulating hormone (TSH) of 3.9 uIU/ml.

**Fig. 2:**
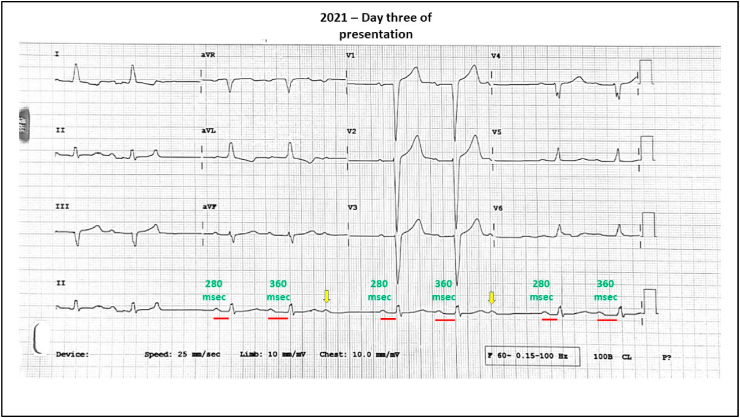
Electrocardiogram on day three showing Mobitz type-I AV block with a serial PR interval of 280 and 360 msec followed by a dropped beat.

**Fig. 3:**
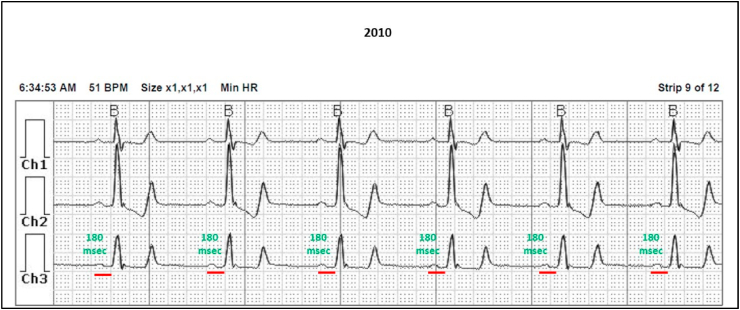
Electrocardiograms of patient from 2010 showing an old LBBB with a PR interval of 180msec.

**Fig. 4:**
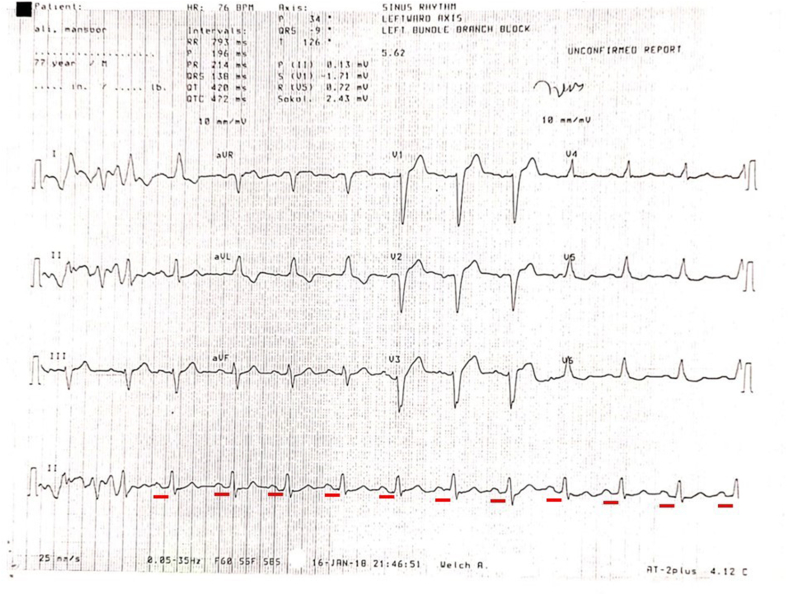
Electrocardiograms of the same patient from 2018 showing an old LBBB with a PR interval of 200 msec.

Echocardiogram was done which showed an ejection fraction (EF) of 60% along with mild mitral regurgitation, moderate tricuspid regurgitation, and moderate pulmonary arterial hypertension. Owing to the hemodynamic stability, and absence of signs of hypoperfusion, temporary pacemaker (TPM) was not inserted. Patient was admitted to the Coronary Care Unit (CCU) of the hospital. Ivabradine was discontinued and patient was observed for any change in rhythm. On day three of CCU admission, patient's rhythm changed to Mobitz type-I AV block with a serial PR interval of 280 and 360 msec followed by a dropped beat (Fig. 2). Older ECGs of same patient were retrieved dating back to 2010 and 2018. The ECG from 2010 showed a normal sinus rhythm with an LBBB and a PR interval of 180 msec (Fig. 3). The ECG from 2018 showed similar findings except for a PR interval of 200 msec (an absolute increase in PR interval of 20 msec over 8 years) (Fig. 4).

**Fig. 5:**
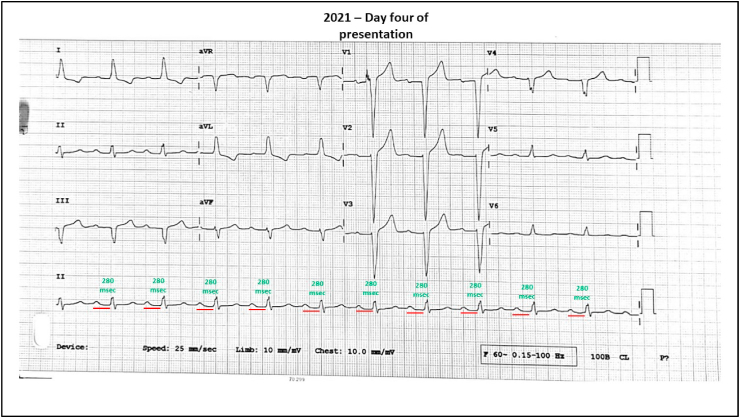
Electrocardiogram on day fourth of presentation showing a normal sinus rhythm with 1:1 P to QRS conduction and a persistent first-degree AV block (PR interval 280 msec).

Patient was further observed and mobilized cautiously with no resultant worsening of AV block. On day four of hospitalization, patient's rhythm changed to a normal sinus rhythm with 1:1 P and QRS conduction (Fig. 5). The PR interval remained 280 msec with an underlying LBBB. Occasionally, patient went into intermittent short-lasting 2:1 AV block.

Possible differentials for an acute AV block were electrolyte imbalance, ivabradine and conduction system disease progression. Disease progression was indeed present due to an increase in first degree AV block. However, the reversibility in P to QRS conduction abnormality pointed towards an acute insult which resulted in a new onset symptomatic bradycardia. Persistence of AV block even after correction of sodium suggests that hyponatremia was unlikely to be the culprit. Our patient was on Ivabradine for last many years, however, again the acuteness of onset goes against the ivabradine being culprit. Hence, it was concluded that COVID-19 vaccination transiently worsened the AV block in our patient. Myocarditis was ruled out because of negative cardiac biomarkers and normal EF.

**Fig. 6:**
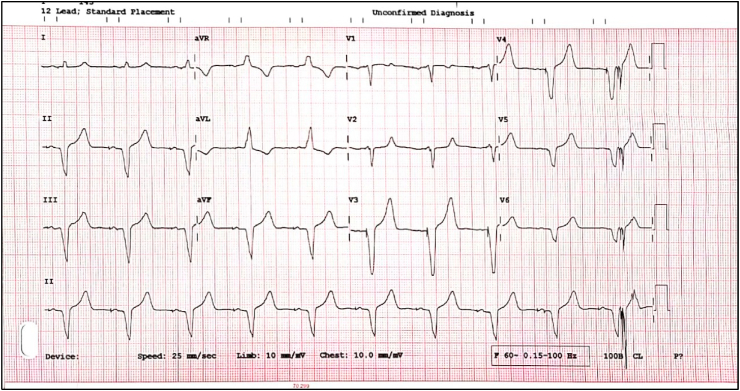
Electrocardiogram showing atrioventricular sequential pacing after permanent pacemaker insertion.

Because of the age, symptomatic documented bradycardia, an underlying AV node (first degree AV block) and infra-HIS disease (LBBB), an expected life expectancy of more than one year, and because of the recurrence of intermittent 2:1 block during the hospital course, a decision to place a dual-chamber permanent pacemaker (PPM) was made. Patient underwent successful device placement (Fig. 6). The procedure was performed by electrophysiologist-on-call in electrophysiology lab of the hospital. Post-procedure, patient was monitored for symptoms. There was no recurrence of symptoms and patient was well-mobilized. He was discharged home the next day, and he followed up in cardiology clinic with improvement in shortness of breath and no recurrence of dizziness or syncope.

## 3 Discussion

In a phase I/II BBIBP-CorV (Sino-pharm) vaccine trial conducted in Henan province of China, no atrioventricular block was reported [9]. However, 29% of the total recipients experienced at least one side effect in first 7 days of inoculation. In another phase I trial, patients with cardiovascular diseases were largely excluded and no arrhythmia was reported [10]. Likewise, another phase I/II trial reported no adverse events pertinent to cardiac arrhythmia [11].

The concept of humoral immune response and AV block is not novel. The association of maternal anti-Ro and anti-La antibodies and congenital complete heart block (CHB) is well known [12]. Like the pattern of maternal antibody immune complex deposition in congenital CHB leading to inflammation and fibrosis in fetal conduction tissue, it is very likely for the inactivated BBIBP-CorV vaccine to have similar pathophysiological basis for causing AV block. In a prospective study of 24 anti-Ro positive pregnant women, 33% of fetuses showed evidence of first-degree AV block, with one fetus showing progression to CHB and another showing shift in degree of AV block from second to first degree. Yet another 6 fetuses, showed resolution of AV block at or soon after birth. Considering this study, it can be interpreted that antibody-associated AV blocks are usually transient (as in our case).

Vaccine-associated AV block has been reported in literature after inoculation of small-pox vaccine in 2003 [13]. In this case, a 56-year-old-man was reported to have first degree AV block and negative cardiac biomarker on day 23 of the vaccination. Likewise, an intermittent incomplete right bundle branch block (RBBB) was also reported after small-pox vaccination in 2009 [14].

The transient nature of AV block in our patient following COVID-19 vaccination can be linked to the pathophysiology of transient and various degrees of heart blocks associated with Lyme carditis. This was evident in an experiment on non-human primates injected with Borrelia burgdorferi strains, whereby an increase in IgG and IgM levels in recipients was observed. There was an accompanying increased deposition of complement-membrane attack complex [15]. The reversible nature of Lyme carditis-AV block is due to the parallelly reduced degree of inflammation over time-course in experimental mouse-models [16]. This stretches our thought process to the possible role of endomyocardial biopsy (EMB) in COVID-19-vaccine associated AV block. EMB is found to demonstrate myocardial lesions other than myocarditis in patients with primary AV block of unknown etiology [17]. EMB, as of now, is not recommended in patients with COVID-19 suspected of having myocarditis [18]. However, like its indication in unexplained early AV blocks [19], EMB can be considered in patients with transient nature of AV block following COVID-19 vaccination. Prognostic significance of EMB in COVID-19 associated AV blocks [20] needs further validation.

Our patient presented with the onset of symptoms 3 days following COVID-19 vaccination. The time scale (within first 7 days of inoculation) between vaccination and worsening of AV block points towards possible association. Additionally, the reversibility of AV block in next few days also points towards an acute insult rather than a chronic irreversible worsening of conduction system tissue. It is likely that patients with an already diseased conduction system are at an increased risk of worsening of AV block following inoculation of the vaccine.

**Fig. 7:**
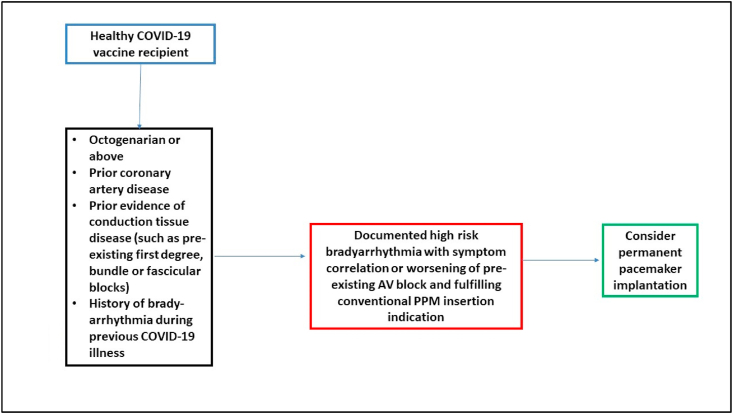
The proposed strategy to identify healthy COVID-19 vaccine recipients who are at increased risk of developing worsening atrioventricular block.

The role of steroids in COVID-19-vaccine associated AV block needs further research [21]. It can be extrapolated from the study that looked at the benefits of antenatal steroids in treatment of congenital CHB whereby, steroids conferred no mortality benefit but significantly downgraded the degree of AV block [22]. Based on our experience in this case, we hereby propose an algorithm to identify healthy COVID-19 vaccine recipients who are at an increased risk of developing worsening AV block. The individualized decision for permanent device placement is re-enforced (Fig. 7).

Given the extensive use of vaccination in general population including patients with ischemic heart diseases, reporting such cases is imperative. Physicians should be cognizant of the possibility of worsening of AV block following vaccination. Watchful waiting strategy can be applied in patients with low-risk baseline ECG whereas an early pacemaker implantation can be considered in patients with high-risk baseline ECG and having an indication for PPM.

## 4 Conclusion

• Patients with underlying existing conduction tissue disease might be at an increased risk of worsening of AV block following inoculation of BBIBP-CorV vaccine.
• Reversibility or intermittency is commonly seen with vaccine- or antibody-associated AV blocks.
• The decision to implant PPM in vaccine-associated AV block should be individualized, as discussed below.
• In patients with absence of an underlying coronary artery or conduction system disease, COVID-19 vaccine associated AV block might be transient and watchful strategy can be employed.
• In presence of an underlying coronary artery or conduction system disease, one must be considerate of the symptom-bradycardia correlation, the degree of underlying block, patient's life expectancy and the overall benefit of device implantation.
• EMB might emerge as a prognostic tool to guide decision for PPM implantation in patients with transient nature of AV blocks following a humoral response.

## Provenance and peer review

Not commissioned, externally peer reviewed.

## Ethical consideration

Written informed consent was obtained from the patient.

## Source of funding

None.

## Author contribution

Pirbhat Shams: Manuscript writing, literature search and final drafting.

Jamshed Ali, Sheema Saadia: literature search and drafting.

Javaid Tai, Aamir Hameed Khan and Fateh Ali Tipoo: Supervision from conceptualization to editing final manuscript.

## Consent

Written informed consent was obtained from the patient for publication of this case report and accompanying images. A copy of the written consent is available for review by the Editor-in-Chief of this journal on request.

## Guarantor

Dr. Javed Tai.

## Declaration of competing interest

None of the authors has any conflict of interests to declare.

## References

[1] Azarkish M., Laleh Far V., Eslami M., Mollazadeh R. (2020). Transient complete heart block in a patient with critical COVID-19. Eur. Heart J..

[2] Eneizat Mahdawi T., Wang H., Haddadin F.I., Al-Qaysi D., Wylie J.V. (2020). Heart block in patients with coronavirus disease 2019: a case series of 3 patients infected with SARS-CoV-2. HeartRhythm Case Rep..

[3] Hosseini Z., Ghodsi S., Hejazi S.F. (2021). Persistent complete heart block in a patient with COVID-19 infection: a case report. SN Comprehens. Clin. Med..

[4] Haddadin F.I., Mahdawi T.E., Hattar L., Beydoun H., Fram F., Homoud M. (2021). A case of complete heart block in a COVID-19 infected patient. J. Cardiol. Cases.

[5] Chinitz J.S., Goyal R., Harding M., Veseli G., Gruberg L., Jadonath R. (2020). Bradyarrhythmias in patients with COVID-19: marker of poor prognosis?.

[6] Lazzerini P.E., Boutjdir M., Capecchi P.L. (2020).

[7] vaccine AUsrrbafC, University/AstraZeneca O. (2021).

[8] Agha R.A., Franchi T., Sohrabi C., Mathew G., Kerwan A. (2020). The SCARE 2020 guideline: Updating Consensus Surgical CAse REport (SCARE) guidelines. Int. J. Surg..

[9] Xia S., Zhang Y., Wang Y., Wang H., Yang Y., Gao G.F. (2021). Safety and immunogenicity of an inactivated SARS-CoV-2 vaccine, BBIBP-CorV: a randomised, double-blind, placebo-controlled, phase 1/2 trial. Lancet Infect. Dis..

[10] Xia S., Duan K., Zhang Y., Zhao D., Zhang H., Xie Z. (2020). Effect of an inactivated vaccine against SARS-CoV-2 on safety and immunogenicity outcomes: interim analysis of 2 randomized clinical trials. Jama.

[11] Keech C., Albert G., Cho I., Robertson A., Reed P., Neal S. (2020). Phase 1-2 trial of a SARS-CoV-2 recombinant spike protein nanoparticle vaccine. N. Engl. J. Med..

[12] Ambrosi A., Wahren-Herlenius M. (2012). Congenital heart block: evidence for a pathogenic role of maternal autoantibodies. Arthritis Res. Ther..

[13] (2003). Update: Adverse Events Following Smallpox Vaccination --- United States.

[14] Sano J., Chaitman B.R., Swindle J., Frey S.E. (2009). Electrocardiography screening for cardiotoxicity after modified Vaccinia Ankara vaccination. Am. J. Med..

[15] Cadavid D., Bai Y., Hodzic E., Narayan K., Barthold S.W., Pachner A.R. (2004). Cardiac involvement in non-human primates infected with the Lyme disease spirochete Borrelia burgdorferi. Lab. Investig.; J. Tech. Methods Pathol..

[16] Saba S., VanderBrink B.A., Perides G., Glickstein L.J., Link M.S., Homoud M.K. (2001). Cardiac conduction abnormalities in a mouse model of Lyme borreliosis. J. Intervent. Card Electrophysiol. : Int. J. Arrhythmias Pacing.

[17] Uemura A., Morimoto S., Hiramitsu S., Hishida H. (2001). Endomyocardial biopsy findings in 50 patients with idiopathic atrioventricular block: presence of myocarditis. Jpn. Heart J..

[18] ESo Cardiology (2020).

[19] Cooper L.T., Baughman K.L., Feldman A.M., Frustaci A., Jessup M., Kuhl U. (2007). The role of endomyocardial biopsy in the management of cardiovascular disease: a scientific statement from the American heart association, the American College of cardiology, and the European Society of Cardiology. Endorsed by the heart failure Society of America and the heart failure association of the European Society of Cardiology. J. Am. Coll. Cardiol..

[20] Wenzel P., Kopp S., Göbel S., Jansen T., Geyer M., Hahn F. (2020). Evidence of SARS-CoV-2 mRNA in endomyocardial biopsies of patients with clinically suspected myocarditis tested negative for COVID-19 in nasopharyngeal swab. Cardiovasc. Res..

[21] Mofors J., Sonesson S.-E., Wahren-Herlenius M. (2020). Effects of maternal medication on long-term outcome in congenital heart block remain to be established. Response to: ‘Comorbidity and long-term outcome in patients with congenital heart block and their siblings exposed to Ro/SSA autoantibodies in utero’ by Satis <em>et al</em&gt.

[22] Michael A., Radwan A.A., Ali A.K., Abd-Elkariem A.Y., Shazly S.A. (2019). Use of antenatal fluorinated corticosteroids in management of congenital heart block: systematic review and meta-analysis. Eur. J. Obstet. Gynecol. Reprod. Biol. X.

